# High correlation of temporal muscle thickness with lumbar skeletal muscle cross-sectional area in patients with brain metastases

**DOI:** 10.1371/journal.pone.0207849

**Published:** 2018-11-29

**Authors:** Johannes Leitner, Sebastian Pelster, Veronika Schöpf, Anna S. Berghoff, Ramona Woitek, Ulrika Asenbaum, Karl-Heinz Nenning, Georg Widhalm, Barbara Kiesel, Brigitte Gatterbauer, Karin Dieckmann, Peter Birner, Daniela Prayer, Matthias Preusser, Julia Furtner

**Affiliations:** 1 Department of Biomedical Imaging and Image-guided Therapy, Medical University of Vienna, Vienna, Austria; 2 Institute of Psychology, University of Graz, Graz, Austria; 3 BioTechMed, Graz, Austria; 4 Department of Medicine I, Medical University of Vienna, Vienna, Austria; 5 Comprehensive Cancer Center, Medical University of Vienna, Vienna, Austria; 6 Computational Imaging Research Lab Department of Biomedical Imaging and Image-guided Therapy, Medical University of Vienna, Vienna, Austria; 7 Department of Neurosurgery, Medical University of Vienna, Vienna, Austria; 8 Department of Radiotherapy, Medical University of Vienna, Vienna, Austria; 9 Department of Pathology, Medical University of Vienna, Vienna, Austria; University of South Alabama Mitchell Cancer Institute, UNITED STATES

## Abstract

**Objectives:**

This study aimed to assess the correlation of temporal muscle thickness (TMT), measured on routine cranial magnetic resonance (MR) images, with lumbar skeletal muscles obtained on computed tomography (CT) images in brain metastasis patients to establish a new parameter estimating skeletal muscle mass on brain MR images.

**Methods:**

We retrospectively analyzed the cross-sectional area (CSA) of skeletal muscles at the level of the third lumbar vertebra on computed tomography scans and correlated these values with TMT on MR images of the brain in two independent cohorts of 93 lung cancer and 61 melanoma patients (overall: 154 patients) with brain metastases.

**Results:**

Pearson correlation revealed a strong association between mean TMT and CSA in lung cancer and melanoma patients with brain metastases (0.733; p<0.001). The two study cohorts did not differ significantly in patient characteristics, including age (p = 0.661), weight (p = 0.787), and height (p = 0.123). However, TMT and CSA measures differed significantly between male and female patients in both lung cancer and melanoma patients with brain metastases (p<0.001).

**Conclusion:**

Our data indicate that TMT, measured on routine cranial MR images, is a useful surrogate parameter for the estimation of skeletal muscle mass in patients with brain metastases. Thus, TMT may be useful for prognostic assessment, treatment considerations, and stratification or a selection factor for clinical trials in patients with brain metastases. Further studies are needed to assess the association between TMT and clinical frailty parameters, and the usefulness of TMT in patients with primary brain tumors.

## Introduction

Sarcopenia is defined as a reduction of skeletal muscle mass and function.[1,2] It was first used to describe age-related muscle mass loss in the elderly population.[1] Secondary causes for sarcopenia are diverse, and include malnutrition, chronic disease, and cancer.[3] Moreover, sarcopenia has an important role in cancer-related cachexia, which has been defined by an international committee as a syndrome, which consists of skeletal muscle mass loss associated with increasing functional impairment that is not reversible by conventional nutrition supplementation.[4] Sarcopenic cancer patients are known to have a poorer outcome with regard to postoperative complications, tumor progression time, and overall survival.[5–6] Dual-energy X-ray absorptiometry (DXA) is an established method to precisely evaluate human body composition.[7] Mourtzakis et al. showed a high correlation between the skeletal muscle mass as assessed by DXA and the skeletal muscle cross-sectional area (CSA) at the level of the third lumbar vertebra obtained by computed tomography (CT) images.[8] Moreover, they presented sex-specific cut-off values to determine sarcopenia for the lumbar CSA adjusted to patient height representing a skeletal muscle index (SMI).[5,9] Based on this study, sarcopenia has been determined by an international consensus as a SMI of ≤ 55 cm^2^/m^2^ for men and ≤ 39 cm^2^/m^2^ for women, although the sex-specific SMI cut-off values vary in the literature.[4,5,8,10] However, in patients with brain metastases, CT examinations at the level of the third lumbar vertebra are not always available. Performing dual-energy X-ray absorptiometry technique or CT scans solely in order to assess or estimate skeletal muscle mass would lead to an increased radiation dose for the patients, as well as additional healthcare costs.

Recently published papers indicate that, in addition to lumbar skeletal muscles, craniofacial skeletal muscles are also useful for the assessment of muscle mass loss.[11,12] This would be advantageous, especially in patients with brain tumors, in which abdominal CT scans are often not available.

Swartz et al. revealed that the cross-sectional area of skeletal muscles at the level of the third cervical vertebra, obtained by head and neck CT images, can assess sarcopenia in head and neck cancer patients.[11] Ranganathan K et al. demonstrated a correlation between the psoas muscle area and temporal muscle thickness (TMT).[12] Moreover, we showed recently that TMT allows survival prediction in patients with newly diagnosed brain metastases.[13]

The purpose of this study was to investigate whether TMT, obtained on routine magnetic resonance (MR) images of the brain correlates with CSA of skeletal muscles, assessed on CT images at the level of the third lumbar vertebra, and thus, may be used to estimated skeletal muscle mass in patients with brain metastases.

## Methods

### Patients

Patients of the Medical University of Vienna with brain metastases from either lung cancer or melanoma between 2002 and 2014 were identified from a brain metastasis database. Inclusion criteria were (a) an MR examination of the brain and an abdominal CT scan of the same patient within one month, and (b) assessable information about a patient`s weight and height within one month of the imaging examination. Lung cancer and melanoma patients were selected for this investigation because those tumor entities are prone to spread to the CNS and do receive CT scans of the abdomen for tumor staging at our institution.[14,15]

We could include 93 lung cancer and 61 melanoma patients in this study (total: n = 154 patients). The study was approved by the ethics committee of the Medical University of Vienna (Vote 1538/2017; 1551/2017). Written informed consent was waived by the ethics committee due to the retrospective design of the study. All patient records used in this study were anonymized before data assessment.

### Analyses of TMT on brain MR images

TMT was measured manually on a picture archiving and communication system (PACS). Measurements were taken perpendicularly to the long axis of the temporal muscle on contrast-enhanced isovoxel 1 x 1 x 1 mm T1-weighted MR images without fat-saturation obtained by 1,5 Tesla or 3 Tesla MRI scanners. The MR plane was oriented axial, parallel to the anterior commissure–posterior commissure line. The measurements were taken at the level of the orbital roof, and the Sylvian fissure was defined as a reference point regarding the anterior-posterior orientation. TMT was evaluated on the left and on the right side separately, in all included patients, by a board-certified radiologist (JF) blinded to the results of CSA on abdominal CT images and to all clinical characteristics. The TMT measurements of each side were summed and divided by two, resulting in a mean TMT per patient.

### Analyses of CSA on abdominal CT images

The CSA of the lumbar skeletal muscles was evaluated on venous-phase CT images of the abdomen. A single slice was manually selected on the level of the third lumbar vertebra where both transverse processes were depicted. The open-source image processing program NIH ImageJ was used for further image analysis.[16] To assess the CSA of the skeletal muscles and exclude visceral organs and adipose tissue at this level, a semi-automated, specific tissue segmentation was performed using the Hounsfield unit thresholds from -29 to + 150.[5,6] Falsely demarcated structures were manually corrected. To calculate the SMI, CSA values were divided by the square of patient height as previously defined in the literature.[5,8]

**Fig 1:**
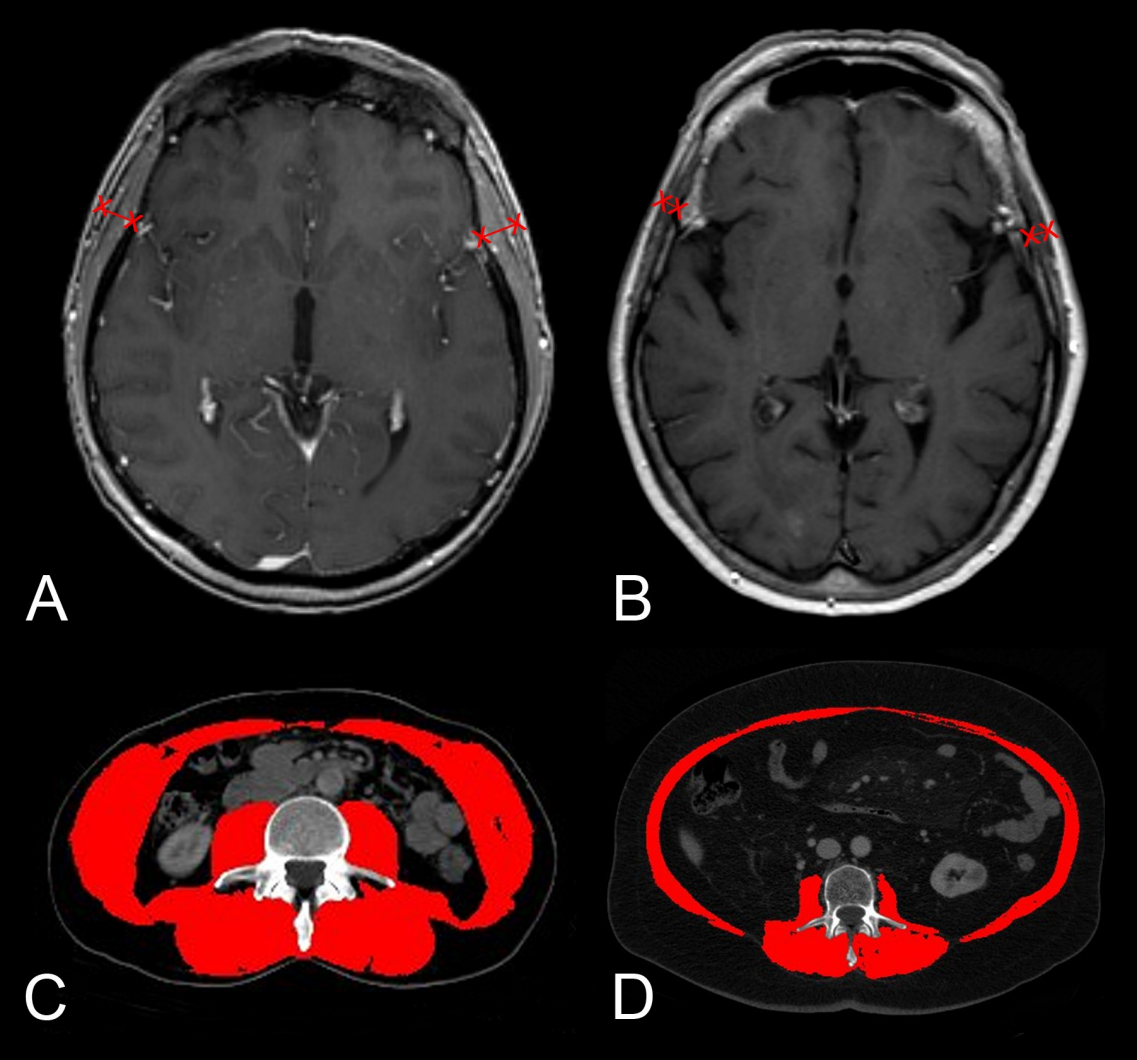
Examples of CSA measurements on CT images (**A**, **C**) and TMT measurements on brain MR images (**B**, **D**) in two melanoma patients with brain metastases. **A, B**, a male patient with an estimated normal skeletal muscle mass (mean CSA = 191.61 mm^2^; mean TMT = 7.3 mm) and **C**,**D**, a female patient with considerable estimated skeletal muscle mass loss (mean CSA = 106.07 mm^2^; mean TMT = 4.5 mm).

Examples of TMT on brain MR images and CSA measurements on CT scans at the level of the third vertebra are provided in **Fig 1.**

### Statistical analysis

Age, TMT, CSA, SMI, and patient weight and height of the two different patient groups were compared using an independent-samples t-test.

To correlate TMT with CSA, SMI, and patient weight and height, a Spearman correlation coefficient was used. A correlation coefficient of (-)0.8 to (-)1 was interpreted as a very strong association, of (-)0.6–0.8 as strong, of (-)0.4 to (-)0.6 as moderate, and of (-)0.2 to (-)0.4 as a low association. There was no association for a correlation coefficient of 0 to (-)0.2. A Mann-Whitney-U-Test was used to reveal differences between sarcopenic and non-sarcopenic patient cohorts. ROC Analysis was used to reveal TMT cut-off measures based on sex-specific SMI cut-off values.

Intra-rater and inter-rater reliability was assessed for the lung-cancer sub-cohort patients (n = 93) using Cronbach's alpha.

Statistical analyses were performed using the Statistical Package for the Social Sciences, Version 24.0 (SPSS, Chicago, Illinois). Parametric testing was used to compare groups.

A two-tailed p-value of < 0.05 was considered statistically significant.

## Results

### Patient characteristics

Overall, 154 patients were available for further analysis, assembled from two independent cohorts that consisted of 93 patients with brain metastases from lung cancer and 61 patients with brain metastases from melanoma. Patient age, sex, height, and weight measures between the two different patient groups did not differ significantly (please see **Table 1** for descriptive measures).

**Table 1:** Descriptive statistics for patient sex, age, height, and weight.

	Melanoma	Lung cancer	p-value
Number of patients	61	93	n.a.
Female	25 (41%)	49 (53%)	n.a.
Male	36 (59%)	44 (47%)	n.a.
Age (mean ± SD, in years)	60.1 (±15.8)	61.5 (±8.3)	0.661
Weight (mean ± SD, in kg)	77.0 (±13.6)	76.3 (±17.0)	0.787
Height (mean ± SD, in m)	1.73 (±0.1)	1.71 (±0.1)	0.123

### Assessment of TMT, CSA and SMI

In lung cancer patients, the mean TMT was 6.1 mm (range, 2.8–9.3 mm), and the mean TMT in melanoma patients was 6.2 mm (range, 2.8–10.9 mm). There was no statistically significant difference in the TMT in these two tumor entities (p = 0.661). The mean TMT differed significantly (p<0.001) between male (7.0 mm; range, 3.8–10.8 mm) and female (5.2 mm; range 2.8–8.5 mm) patients in both study cohorts.

The mean CSA was 130.1 cm^2^ (range, 65.6–208.0 cm^2^) in lung cancer patients and 136.5 cm^2^ (range, 78.8–205.3 cm^2^) in melanoma patients. No statistical difference with regard to the CSA was observed in lung cancer or melanoma patients (p = 0.221). However, the mean CSA showed a significant sex-specific difference (p<0.001), with a mean CSA of 106.10cm^2^ (range, 65.6–138.9 cm^2^) in female and 154.2 cm^2^ (range, 94.13–208.0 cm^2^) in male patients of both study cohorts.

The mean SMI in lung cancer patients was 43.96 cm^2^/m^2^ (range, 24.70–65.85 cm^2^/m^2^) and the mean SMI in melanoma patients was 45.20 cm^2^/m^2^ (range, 27.28–63.35 cm^2^/m^2^). No statistical difference with regard to the SMI was observed in lung cancer patients and melanoma patients (p<0.359). The mean SMI differed significantly (p<0.001) between male (48.51cm^2^/m^2^, range, 27.64–65.85 cm^2^/m^2^) and female (39.46 cm^2^/m^2^, range 24.70–58.13 cm^2^/m^2^) patients in both study cohorts. For detailed information see also **S1 Table.**

After dividing the whole study cohort in sarcopenic (male, n = 65; female, n = 35) and non-sarcopenic (male, n = 20; female, n = 34) patients using a sex-specific cut-off SMI value (39cm^2^/m^2^ for female patients and 55 cm^2^/m^2^) the associated TMT values differed significantly between sarcopenic and non-sarcopenic male (p = 0.004) and female (p = 0.001) patients, respectively.[4]

### Correlation of TMT with CSA and SMI

**Fig 2:**
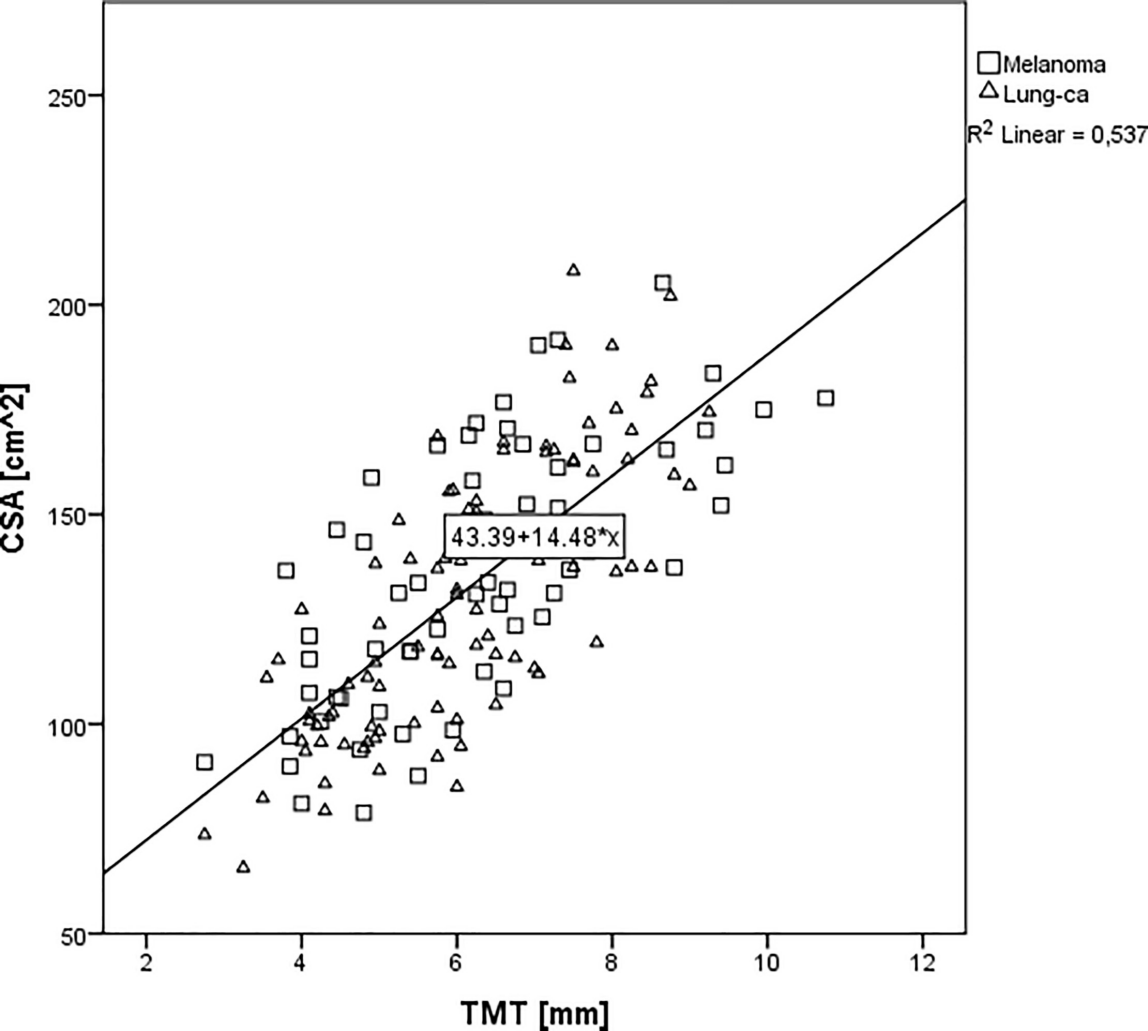
Correlation between CSA of the skeletal muscles at the third lumbar vertebra and the mean TMT values in melanoma (☐) and lung cancer (Δ) patients with brain metastases.

**Fig 3:**
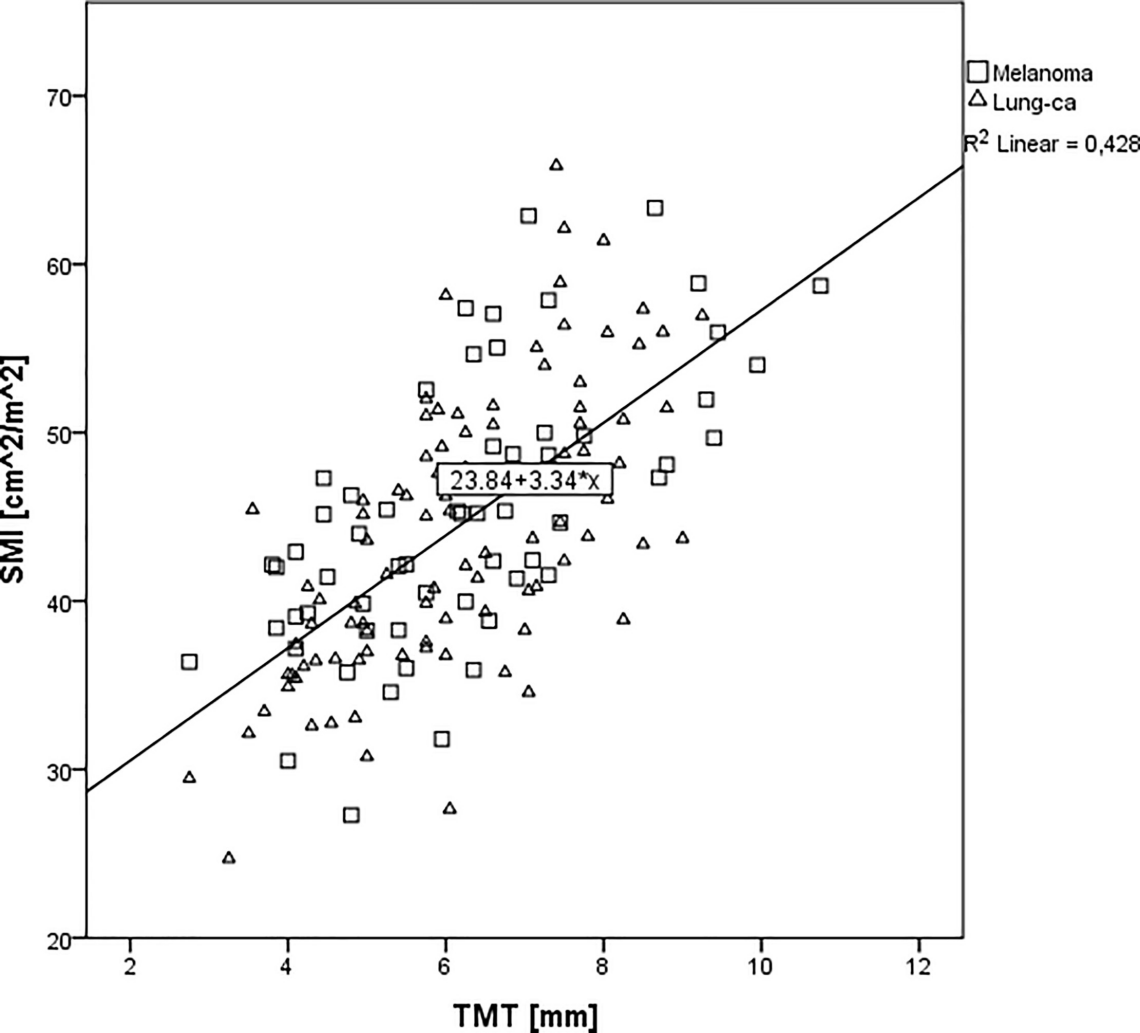
Correlation between SMI values and mean TMT values in melanoma (☐) and lung cancer (Δ) patients with brain metastases.

The correlation between TMT and CSA as well as TMT and SMI in melanoma and lung cancer patients with brain metastases is shown in **Fig 2 and Fig 3**.

The mean TMT showed a highly significant correlation with CSA in both groups (Spearman correlation coefficient 0.733; p<0.001), as well as in the melanoma subgroup (Spearman correlation coefficient 0.692; p<0.001) and lung cancer subgroup (Spearman correlation coefficient 0.767; p<0.001) separately.

The association of mean TMT with SMI values was also highly significant in the melanoma subgroup (Spearman correlation coefficient 0.650; p<0.001) and in the lung cancer subgroup (Spearman correlation coefficient 0.662; p<0.001) resulting in an overall Spearman correlation coefficient between TMT and SMI of 0.655; p<0.001 in both cohorts.

### Correlation of TMT with clinical parameters

The Pearson correlation revealed a moderate association between patient height and TMT measurements in both study groups (Spearman correlation coefficient 0.504; p<0.001). Patient weight showed a low correlation with TMT in the whole study population (Spearman correlation coefficient 0.385; p<0.001). Furthermore, a low negative correlation was detected between TMT and the age at diagnosis of brain metastasis for all patients included in this study (Spearman correlation coefficient –0.246; p = 0.002).

### Assessment of inter- and intra-rater agreement

Intra-rater and intra-rater reliability was assessed for the lung-cancer sub-cohort patients (n = 93) using Cronbach's alpha resulting in a next to perfect intra-rater agreement (left TMT 0.959; right TMT 0.975) and intra-rater agreement (left TMT 0.917; right TMT 0.94).

## Discussion

Sarcopenic cancer patients are known to have an impaired outcome with regard to toxicity when treated with chemotherapy, more postoperative complications, as well as poorer overall survival.[5,6,17,18] Therefore, additional information about skeletal muscle mass loss at the time of disease diagnosis, in combination with other clinical parameters, could influence individual treatment decisions or patient selection and stratification in clinical trials. Therefore, we investigated whether TMT obtained on routine MR images of the brain correlates with CSA of the skeletal muscles at the level of the third lumbar vertebra and SMI values, through the evaluation of CT images in two independent cohorts of melanoma and lung cancer patients with brain metastases to establish a new parameter estimating skeletal muscle mass on brain MR images. The present study revealed a high correlation between the lumbar skeletal muscle area and TMT as well as SMI values and TMT. Moreover, TMT values differed significantly between sarcopenic and non-sarcopenic male (p = 0.004) and female (p = 0.001) patients, respectively, which indicates that TMT can be used to estimate skeletal muscle mass loss and may be a surrogate parameter for sarcopenia.

Previously published findings have already revealed that, in addition to abdominal skeletal muscles, the craniofacial skeletal muscles are also useful for the estimation of muscle mass loss. Swartz et al. showed that the CSA of the skeletal muscles at the level of the third cervical vertebra (including paravertebral muscles and the sternocleidomastoid muscles) highly correlate with the CSA of the skeletal muscles at the level of the third lumbar vertebra and thus, they suggested that also cervical skeletal muscles can be used to estimate the skeletal muscle mass in head and neck cancer patients.[11] Kilgour et al. used the CSA of neck muscles at the level of the second cervical vertebra to estimate age-related muscle mass loss on brain MR examinations.[19] However, in most routine MR scans of our study population, these muscles were depicted only partially or not at all. Therefore, we chose the temporal muscle for skeletal muscle mass estimation on routine brain MR images because it is one of the few muscles that are depicted on routine brain MR images in its full extent. Furthermore, we did not assess a CSA but rather a one-dimensional measurement to avoid time-consuming volume or plane segmentation. In line with previously published data we could show an almost perfect inter-rater and intra-rater agreement, using predefined anatomical landmarks, and are very simple and fast to obtain, at approximately 30 seconds per patient, which makes this technique easily includable in the daily clinical workflow.[13] In contrast, for the CSA assessment, the images had to be exported to another workstation to use a semi-automatic segmentation program (NIH ImageJ) with additional manual correction subsequently, in most cases, which resulted in an overall acquisition time for CSA of approximately 25 minutes.[16]

TMT measurements were correlated with patient characteristics to investigate the influence of patient age, height, or weight. Age and weight of the melanoma and lung cancer patients with brain metastases showed only a low correlation with TMT, and are, therefore, expected not to have a major impact on TMT values. First, these findings demonstrate that, in cancer patients, the muscle mass loss is more apparent compared to the known sarcopenic state of aging alone.[1] Second, the weak correlation between weight and TMT may be due to the fact that patient weight is not able to differentiate between fat and lean body mass. Therefore, it might be assumed that, in contrast to patient weight, TMT is more likely to detect sarcopenic obesity.[5] The mean TMT and CSA differed significantly between male and female patients in both study cohorts. This is attributable to the sex-specific body composition and comparable to results in previously published papers, which also found significant sex-specific differences in mean TMT and CSA values in cancer patients.[13]

Previously published data revealed that height showed the strongest correlation with skeletal muscle mass and strength among body antropometric measures.[20] These findings are in line with our results although TMT measurements showed only a moderate association with patient height. In contrary to indices that estimate skeletal muscle mass based on trunk and limb muscle, height is hardly supposed to affect cranio-facial muscles and therefore we did not adjust TMT by height when comparing individuals of different size.[20, 21] Moreover, the moderate correlation between TMT and patient height is also the reason for the slightly lower correlation of TMT and SMI (CSA/patient height in m^2^) in comparison to TMT and CSA.

Although, TMT values differed significantly between sarcopenic and non-sarcopenic patients no suitable TMT cut-off measure could be defined based on a ROC analysis using sex-specific SMI cut-off values. Furthermore, sex-specific SMI cut-off values vary in the literature with a range from 41,1 cm^2^/m^2^ to 39 cm^2^/m^2^ and from 43,7 cm^2^/m^2^ to 55 cm^2^/m^2^ in female and male patients, respectively.[4,5,10]{Bibliography} Therefore, further prospective studies are needed to correlate TMT with other clinical frailty parameters of the patients in order to define sex-specific cut-off values for the determination of sarcopenia using TMT in brain tumor patients.

A potential limitation of this study is that the assessed diameter of the temporal muscle was relatively small. Therefore, accurate compliance with the use of predefined anatomical landmarks is all the more important to guarantee high measurement accuracy as already shown in a previously published manuscript.[13] Moreover, TMT measurements could be biased by partial volume artefacts. To minimize partial volume artefacts only patients with isovoxel 1 x 1 x 1mm MR images had been included in this study.

However, the temporal muscle has been chosen because it is one of the few muscles that are depicted on routine brain MR images in its full extent. This seemed essential to us to exclude any circumstances, which could influence muscle thickness (e.g. postoperative edema or muscle atrophy).

Another limitation is the retrospective design of this study. As a result, muscular strength or patient frailty could not be considered. To investigate the correlation of TMT with functional parameters (for example, grip strength), and to determine sex-specific cut-off values for a diagnosis of sarcopenia, further prospective studies are needed. However, due to the strong correlation of TMT and CSA of the skeletal muscles of the third lumbar vertebra, our data provide evidence that TMT is a useful parameter with which to estimate skeletal muscle mass in brain metastasis patients. This should also be evaluated to determine whether this is also applicable to other brain tumor types, such as gliomas. Furthermore, TMT could be influenced by oral or dental disease.[22] To overcome this problem, TMT values were measured on both sides and divided by two to calculated the mean TMT value for each patient and reduce dental or oral related muscle changes as far as possible.

We conclude that skeletal muscle mass can be estimated on routine brain MR images in brain metastasis patients and may be used as an alternative to abdominal CT scans. This would be an advantage for patients where abdominal CT scans are not available to reduce patient radiation dose, as well as health costs, and may help in treatment or clinical trial selection. However, further prospective studies designed to correlate TMT with other clinical frailty parameters are required.

## Supporting information

S1 Table Detailed description of TMT, CSA and SMI values. (PDF)

## Abbreviations

CSA: cross-sectional area
CT: computed tomography
MRI: magnetic resonance imaging
SMI: skeletal muscle index
TMT: temporal muscle thickness
