# Supplementary material for: High correlation of temporal muscle thickness with lumbar skeletal muscle cross-sectional area in patients with brain metastases
Source: PLoS One. 2018 Nov 29;13(11):e0207849. doi: 10.1371/journal.pone.0207849 (PMC6264824; doi:10.1371/journal.pone.0207849)
Supplement: S1 Table — (PDF) [file pone.0207849.s001.pdf]

S1 Table. Detailed description of TMT, CSA and SMI values

	Male (n= 85)		Female (n=69)		
	**mean**	**SD**	**mean**	**SD**	**p**
**TMT** (in mm)					
All	6.97	1.47	5.17	1.19	$4.3147*10^{-14}$
Melanoma	6.96	1.70	5.19	1.18	0.000034
Lung-Cancer	6.98	1.23	5.16	1.20	$1.6735*10^{-10}$
**CSA** (in cm^2)					
All	154.20	23.61	106.10	16.97	$2.991*10^{-31}$
Melanoma	156.29	21.84	108.03	16.47	$3.0222*10^{-13}$
Lung-Cancer	152.67	24.95	105.00	16.41	$6.8333*10^{-18}$
**SMI** (in cm^2/m^2)					
All	48.51	7.54	39.46	5.88	$1.2068*10^{-13}$
Melanoma	49.03	7.01	39.70	5.52	$7.0325*10^{-7}$
Lung-Cancer	48.13	7.95	39.32	6.13	$5.3962*10^{-8}$
